# Functional Foods, Nutraceuticals and Probiotics: A Focus on Human Health

**DOI:** 10.3390/microorganisms10051065

**Published:** 2022-05-21

**Authors:** Morayma Ramírez Damián, Naima G. Cortes-Perez, Erika T. Quintana, Alicia Ortiz-Moreno, Cynthia Garfias Noguez, Carlos Eugenio Cruceño-Casarrubias, María Elena Sánchez Pardo, Luis G. Bermúdez-Humarán

**Affiliations:** 1 Instituto Politécnico Nacional, Escuela Nacional de Ciencias Biológicas—Campus Zacatenco, Unidad Profesional Adolfo López Mateos, Zacatenco, Av. Wilfrido Massieu 399, Colonia Nueva Industrial Vallejo, C.P., Alcaldía Gustavo A. Madero, Mexico City 07738, Mexico; morayma.ramirez@outlook.com (M.R.D.); ortizalicia@hotmail.com (A.O.-M.); cgarfez@gmail.com (C.G.N.); charlyska19@gmail.com (C.E.C.-C.); 2 INRAE, Université Paris-Saclay, AgroParisTech, UMR 0496, 78350 Jouy-en-Josas, France; naima.cortes-perez@inrae.fr; 3 Instituto Politécnico Nacional, Escuela Nacional de Ciencias Biológicas—Campus Santo Tómas, Unidad Profesional Lázaro Cárdenas, Prolongación de Carpio y Plan de Ayala s/n, Colonia Santo Tómas C.P., Alcaldía Miguel Hidalgo, Mexico City 11340, Mexico; erika_quintana@hotmail.com; 4 INRAE, Université Paris-Saclay, AgroParisTech, Micalis Institute, 78350 Jouy-en-Josas, France

**Keywords:** functional foods, probiotics, nutraceuticals, lactic acid bacteria, microbiota

## Abstract

Functional foods are classified as traditional or staple foods that provide an essential nutritional level and share potentially positive effects on host health, including the reduction of disease by optimizing the immune system’s ability to prevent and control infections by pathogens, as well as pathologies that cause functional alterations in the host. This chapter reviews the most recent research and advances in this area and discusses some perspectives on what the future holds in this area.

## 1. Introduction

**Figure 1:**
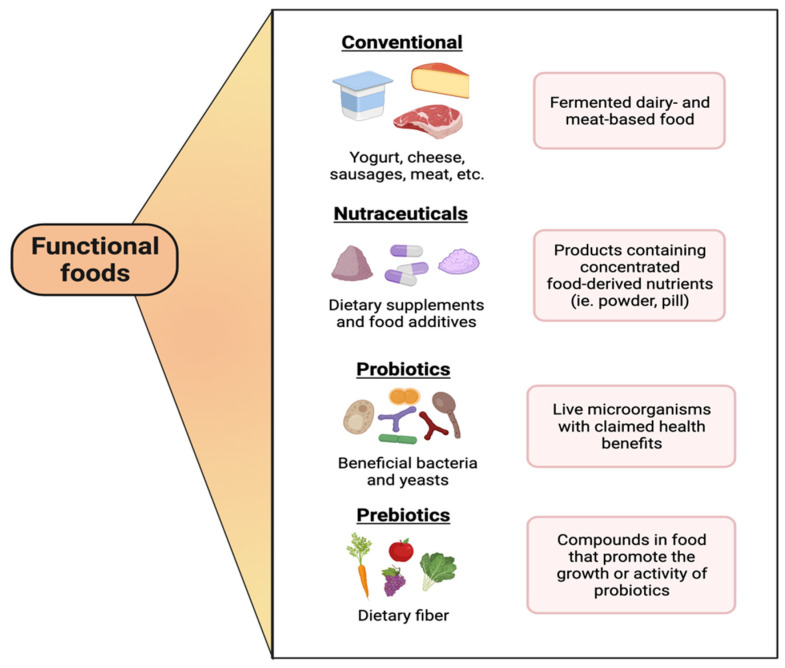
Main types and components of functional foods. This figure was created with biorender.com (accessed date: 26 April 2022).

Although the concept of functional foods has been defined on several occasions, there is no internationally accepted definition of this term [1]. Also, despite efforts to regulate, there is no scientific consensus to distinguish between functional foods, nutraceuticals, and dietary supplements [2,3]; however, experts generally agree that functional foods contain ingredients (including live microorganisms, such as probiotics, see below) that provide health benefits in addition to the basic nutritional components of the food itself. Here we present a general overview of these concepts without going into the details of specific regulations of each region or country. Thus, functional foods can be natural or processed foodstuffs of basic consumption in the diet that provide an essential nutritional level and also share potentially positive effects on the host’s health, including the reduction of diseases by optimizing the capacity of the immune system to prevent and control infections by pathogens, as well as pathologies that cause functional alterations in the host [4]. Functional foods have been described to modify physiological mechanisms at the gastrointestinal tract (GIT) level by increasing biochemical parameters and improving neuronal functions [5]. Some of the main types of functional foods, besides fermented conventional ones, include nutraceuticals, probiotics, prebiotics, and synbiotics (which are a mixture of probiotics and prebiotics) (Figure 1).

Nutraceuticals, unlike conventional diets, have been described as foods (or part of a food) combining nutritional and pharmaceutical effects (i.e., providing medical or health benefits, including the prevention and/or treatment of a disease). Hence, nutraceuticals include any compound of natural origin that positively effects the human body accompanied by a nutritional value [6]. Within the most common functional foods, nutraceuticals are frequently present. It has been proposed that some groups of molecules, macronutrients, and micronutrients, found in these products are responsible for the beneficial effects observed in the host: these include polyphenols, alkaloids, terpenoids, unsaturated fatty acids, vitamins A, B6, B12, C, D, E, folate, and some trace elements, such as zinc, iron, selenium, magnesium, and copper [7]. In addition, within functional foods, we can also cite polyunsaturated fatty acids (PUFAs) and antioxidants (such as β-carotenes and lycopene), which have also been found in nutraceuticals [4,8]. It is important to note that according to this definition of nutraceuticals and the regulatory proposal made by Zeisel et al. [2], probiotics and prebiotics would also be part of nutraceuticals if they are present in pill form and not in a food matrix [2]. However, for practical reasons, we present them separately here.

Food-derived bacteria (such as probiotics) secrete active compounds that are by-products of their metabolism, such as essential vitamins, antioxidants, and short-chain fatty acids (SCFAs), which have a direct impact on host health [9,10]. Probiotics have been defined by the World Health Organization (WHO) since 2011; however, the concept was re-examined in 2013 by a group of experts to agree on “live microorganisms that, when administered in adequate amounts, confer a health benefit on the host” [3,11]. It has been shown that more than different bacterial species, coming mostly from our diet (e.g., functional food), can inhabit the human GIT. Among them, we find mainly species belonging to lactobacilli, *Bifidobacterium*, and *Enterococcus* [12,13].

## 2. Proposed Mechanisms by Which Functional Foods Exert Their Beneficial Effects

**Figure 2:**
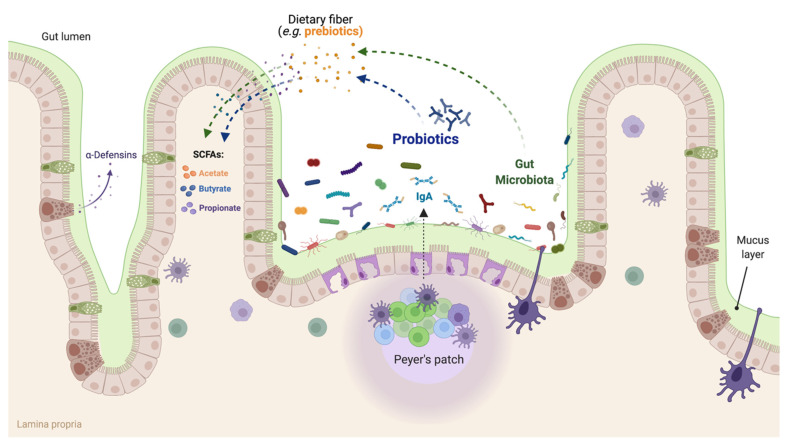
Some of the proposed mechanisms by which probiotics exert their beneficial effects. This figure was created with biorender.com (accessed date: 26 April 2022).

It has been possible to propose some mechanisms of action of the compounds that make up functional foods using metagenomics and metabolomics technologies. Some of these mechanisms undoubtedly include SCFAs (e.g., acetate, butyrate, and propionate) (Figure 2), which display different immunomodulatory activities [14], omega-3 fatty acids and glutamine that act as antioxidants preventing or delaying oxidation and peroxidation of diverse vital cellular components; for example, they neutralize free radicals and prevent cell damage and oxidative stress of the endoplasmic reticulum, decreasing the damage caused by inflammation in specific pathologies [15].

Probiotics are part of functional foods and are proposed as one of the main mechanisms providing beneficial effects. It has been shown that the activity of the intestinal microbiota plays an essential role in the energy supply of the host [16]. Therefore, supplementing the diet with live microorganisms, such as probiotics, helps the intestinal microbiota with this important role. Probiotic bacteria also protect the mucosal barrier of the intestinal epithelium against pathogenic microorganisms (Figure 2) [9]. For instance, the production of mucin and the reduction of intestinal permeability by lactic acid bacteria (LAB) prevent the proliferation of pathogens and their access to intestinal epithelial cells. The surface coat protein of *Lactobacillus crispatus* and *Lactobacillus helveticus* prevents the in vitro adhesion of pathogenic strains of *E. coli* O157:H7 and *Salmonella typhimurium* (usually transmitted to the host by ingestion of contaminated food) to Hela, HEp-2, and T84 cells [17]. Additionally, the production of lactate and acetate as a product of lactic fermentation carried out by LAB is another defense mechanism as this phenomenon creates an acidic microenvironment that inhibits the growth of pathogens [18].

Probiotic bacteria can also synthesize vitamins, immunomodulatory proteins, and a broad range of peptides with inhibitory activities, such as class II peptide bacteriocins and class III bacteriolysins. For example, *Limosilactobacillus reuteri* produces a peptide called reuterin, a potent antibacterial, antifungal, antiviral, and antiprotozoal peptide [19]. In addition, strains that produce these antimicrobial compounds (or the peptides directly) are commonly used in the food industry as preservative agents [19]. Furthermore, many probiotic bacteria can adhere to the mucosa of the GIT and stimulate the immune system. Indeed, certain strains of probiotics have been shown to stimulate Peyer’s patches, which can take up pathogens and release immune system cells, such as follicular dendritic cells, and stimulate cytotoxic CD8+ T lymphocytes, manifesting decreased cytokine expression, systemic inflammation, cell proliferation, and increased apoptosis of damaged cells [20].

Fermented foods, such as some types of milk (which are widely consumed beverages), have been proven to be good functional foods because they contain probiotic bacteria, such as *L. helveticus*, capable of producing factors during milk fermentation that stimulate the production of the enzyme calcineurin. Calcineurin is responsible for activating the transcription of interleukin-2 (IL-2) and participating in the stimulation of the growth and differentiation of T lymphocytes [21].

## 3. Different Classes of Probiotic Products

Currently, the food industry has seen significant growth in developing functional foods, mainly by applying probiotic bacteria as ingredients in the formulation of its products and as food additives [22].

LAB, a group of microorganisms within which several probiotic strains are found today, has been present in many foods since humans began consuming foods whose manufacturing processes include fermentation. Therefore, these have been part of this traditional food manufacturing process, with lactic acid fermentation being the main metabolic pathway. However, over time, two main sources of LAB isolation (mainly used as probiotics) have emerged as classified by [23]: one from dairy products and the other from non-dairy products. Within dairy-based products, Vijaya Kumar et al. [24] include sour buttermilk, unfermented goat milk drink, sour milk, ice cream, and yogurt as isolate sources. Within non-dairy, two categories are included, fermented and non-fermented, including in both groups cereals, fruits, vegetables, meats, and fish [25].

### 3.1. Dairy-Based Functional Foods Containing Probiotics

Several LAB have been isolated from fermented dairy products and have been shown to have a beneficial effect. Therefore, from these fermented dairy products, many foods with probiotic activity have been developed, such as yogurt, kefir, or dairy serums containing lactobacilli and *Bifidobacterium* [26].

#### 3.1.1. Fresh and Fermented Milks

The probiotic properties of dairy products such as milk have been explored by studying the bacteria that these products contain. Hurtado-Romero et al. [27] studied the probiotic properties, prebiotic fermentability, and GABA production capacity of microorganisms isolated from Mexican milk kefir grains obtained in the Guadalajara region of Jalisco, Mexico. These same authors performed a grouping for dairy functional food applications, identifying strains of bacteria belonging to the genera *Lactococcus*, lactobacilli, *Leuconostoc*, and *Kluyveromyces* in milk.

The use of functional foods supplemented with probiotic bacteria has also been evaluated in a large number of studies in people suffering from a disease in order to propose a treatment based on a diet containing functional foods, as demonstrated by Zepeda-Hernández and collaborators [28] when they found that a diet based on the consumption of dairy products that included yogurt, fermented milk, and cheese, added with potential probiotic strains of *Lactobacillus acidophilus*, *Lacticaseibacillus casei*, *Bifidobacterium*, and prebiotics or symbiotics (i.e., a mixture of both: probiotics and prebiotics), improved the symptoms of type 2 diabetes mellitus by reducing oxidative stress, inhibiting pro-inflammatory markers, and reducing intestinal dysbiosis.

#### 3.1.2. Yogurt

In the food industry, the high demand for probiotic-based yogurt production is growing every day. Each probiotic strain offers different and specific health benefits, as proposed by Mani-Lopez et al. [29], which studied the probiotic viability and storage stability of different types of yogurts and fermented milk prepared with various mixtures of LAB, mainly of the genera *Lactobacillus delbrueckii* ssp. *bulgaricus* and *Streptococcus thermophilus*.

#### 3.1.3. Cheese

Cheese is a food product made from milk curd. In many cases, the cheese’s flavor, taste, consistency, texture, and appearance of the cheese depend on the fermentation carried out by the microorganisms, such as LAB, as previously reported by Falfán-Cortés et al. [30]. They analyzed the characterization and evaluation of the in vitro and in situ probiotic potential of *Lacticaseibacillus paracasei* isolated from Tenate Cheese, proposing this strain as a possible probiotic with a high antagonistic potential against pathogens in food technology.

Artisanal cheeses from various regions of Mexico were studied by Özkan et al. [31]. This group screened lactobacilli strains from artisanal cheeses from Tulum (through molecular and in vitro probiotic characteristics), which had been prepared by covering them with Turkish goatskin produced by nomads. Thus, they identified a strain of *Lactiplantibacillus plantarum* isolated from cheese from the Tulum region and proposed it as a promising probiotic candidate and suitable for use in various fermented foods.

#### 3.1.4. Other Dairy Products

Probiotic bacteria have been isolated from various products of dairy origin, such as butter, from which lactobacilli strains have been isolated with antagonistic capacity against pathogens such as *Salmonella* and *Listeria* [32]. When strains of *Lacticaseibacillus casei* subsp. *casei* AB16−65 and *Lactobacillus maltaramicus* AC 3−64 16 are added to butter, they impact the reduction of cholesterol content and saturated fatty acids, decreasing the cardiovascular damage caused by the continuous consumption of butter as reported by Aloğlu and Öner [33].

In addition, *Pediococcus acidilactici* strain LRCC5307 has been shown to be a LAB that assimilates cholesterol well. Therefore, its addition to butter has been proposed to propose a functional low-cholesterol butter food [34].

Functional dairy foods have also been proposed from cream, with high health benefits, as demonstrated by Ekinci F et al. [35]. For instance, they added *B. bifidum*, *L. acidophilus*, *S. thermophilus*, and *Lactobacillus delbrueckii subsp. bulgaricus* to a cream supplemented with sunflower oil, hazelnut oil, and soybean oil, allowing a fermentation period, resulting in a high content of capric, butyric, and caproic acid, acids that have been shown to provide important health benefits to the host.

Ice cream has also begun to be used as a somewhat more unusual source of probiotic isolation and supplementation. Such is the case of the addition of *L. acidophilus* strain ATCC 4356 to ice cream to test the survival conditions of the strain, as well as its beneficial effect on fat reduction (from high-fat ice cream processing ingredients), proposing thus a more health-promoting low-fat ice cream [36].

### 3.2. Plant-Based Products Containing Probiotics

It has been proposed that there is a broad relationship between food intake, diet, and the gut microbiota [37]. Studies have shown that the intake of vegetables, which contain high levels of cellulose (as their cell wall), can act as a source of nutrients for LAB, fulfilling the function of a symbiotic and increasing their potential as probiotics within the intestinal microenvironment. Such is the case of kimchi, a traditional food from Korea, whose elaboration is through the fermentation of vegetables with probiotic bacteria such as *Latilactobacillus sakei*, *Leuconostoc citreum*, *Leuconostoc gasicomitatum*, *Leuconostoc gelidum*, and *Levilactobacillus brevis* that perform lactic acid fermentation. Additionally, it has been proven that when adding vegetables such as cabbage, the fermentation parameters increase, producing lactic acid in greater quantity leading to a more significant pH change in the medium [38]. This leads to proposing a possible symbiotic based on vegetables and LAB in functional food.

The dynamic nutritional content and correlation of bacterial communities such as *Weissella*, *Sphingomonas*, *Leuconostoc*, *Pediococcus*, and *Psychrobacterse* strains and their relationship in the production of metabolites in *Raphanus sativus* (a type of radish rich in proteins, glucosinolates, flavonoids, β-carotene, and minerals), which, when treated as a fermented pickle and supplemented with wheat bran, makes it possible to be used as a synbiotic in food preservation and increase its nutritional content [39].

### 3.3. Fruit-Based Products Containing Probiotics

Fruit juices (100% fruit content), nectars (25–99% fruit), and juice drinks (up to 25% fruit) are widely consumed, and added presentations with probiotic bacteria have been suggested. Since it has been proven that the addition of probiotics produces bioactive compounds through the fermentation of sucrose and sugars in the fruit, this classifies them as functional foods by enhancing the antioxidant capacities of both the fruit and the intestinal tract of the individual consuming them [40].

*Lactobacillus delbrueckii subsp. bulgaricus*, *S. thermophilus*, *Saccharomyces cerevisiae*, and *Saccharomyces boulardii* strains have been used mainly used. This proves that their addition to fruit juices favors the intestinal absorption of calcium, iron, and magnesium, as well as intervening in the supply of ascorbic acid (vitamin C) and promoting a protective effect. Studies on the bioavailability and bioaccessibility of these compounds, as well as their relationship with the survival and productivity of probiotic bacteria, are still scarce [41].

The isolation of LAB from corn has also been carried out. In a report study carried out by Septembre-Malaterre et al. [42], they studied several fruits and vegetables and their relationship with lactic fermentation, analyzing the sources of nutritional compounds containing bioactive molecules such as phenolic compounds, flavonoids, bioactive peptides, and phytochemical content.

They concluded that probiotic bacteria, when interacting with fruits rich in β- and α-carotenes such as carrot, pumpkin, mandarin, orange, mango and vegetables, act as symbionts promoting changes in bioactive compounds generating SCFAs, polysaccharides of high nutritional value and bioactive peptides. This additionally proves the reduction of sugar content and that the anti-nutritional phenolic compounds are converted into molecules of high added biological value, turning these fruits and vegetables into functional foods [43].

### 3.4. Cereal-Based Products Containing Probiotics

One of the fields of application of LAB is the fermentation of cereals. The main products obtained in this way are traditional drinks and other traditional fermented foods based on cereals [44,45], but also products from the bakery and pastry sector called sourdoughs. These ecosystems are mixtures of flour and water, composed of a combination of yeast and LAB. Thus, sourdoughs are used in the manufacturing of many products such as sourdough bread, buns, panettone, etc. [46,47]. The LAB involved in these fermentations come mainly from the raw materials, such as cereals (surface microbiota of the seeds). They can also be contributed by the ambient air and the different working environments along the cereal processing chain. The LAB most commonly identified in traditional sourdoughs and fermented beverages were lactobacilli, *Leuconostoc*, *Weissella*, and *Pediococcus*. In addition, other less common genera such as *Streptococcus*, *Enterococcus*, and *Lactococcus* are also present.

Today, the insights provided by traditional fermented grain products could help develop new probiotic products for the food industry, which could help when lactose intolerance and cholesterol content are inconvenient, when people refuse to eat dairy products for particular reasons or when dairy products are not available. Indeed, some of the probiotic functions attributed to some LAB strains isolated from cereal products include immune stimulation, pathogen exclusion, production of bioactive substances, and overall gut health [48,49,50]. For example, sourdough has been used since ancient times for the production of rye and wheat bread, and its use has led to the improvement of the quality, nutritional properties, and shelf life of sourdough-based bread [51].

### 3.5. Meat-Based Products Containing Probiotics

The meat industry is constantly researching new alternatives for livestock feeding. For the production of meat with high nutritional value, mainly in cattle, the association of ionophores, yeasts, and probiotics (such as some strains of lactobacilli and *S. cerevisiae*) is present. Indeed, a cattle feed formulation alters the abundance of ruminant microbial species by promoting colonization of the animal’s intestinal tract, improving nutrient absorption, and promoting the growth of cattle muscle mass. This demonstrates that probiotics added to the feed improve animal meat quality by reducing the number of pathogenic bacteria such as strains of *Prevotella ruminicola*, *Selenomonas ruminantium*, and the commensal *Streptococcus bovis* that affect nutrient absorption [52].

A study conducted by Da Silva-Marques et al. [53] found that protein supplementation improves some non-harmful fermentation parameters in the rumen microbial populations of cattle destined for meat. They found a decrease in colonization by *Butyrivibrio fibrisolvens* and an increase in the population of *Fibrobacter succinogenes*, *Ruminococcus albus*, *Ruminococcus flavefaciens*, and *Methanogen archaea*, demonstrating that bacteria with possible probiotic activity and cellulite activity improve the quality of the meat product.

## 4. Fermented Foods Containing Live Probiotic Cultures

Fermented food products are foods and beverages that, in their preparation, include a process in which controlled microbial growth is produced, involving the conversion by enzymatic reactions of the macronutrients that make up the food and its components [54]. Fermented foods include many traditionally consumed beverages and foods such as kefir, kombucha, sauerkraut, tempeh, natto, miso, kimchi, and sourdough bread [54]. As an example, in the production of kefir, a varied and extensive list of microorganisms is used, such as the yeasts *Kluyveromyces marxianus*, *S. cerevisiae*, *Saccharomyces unisporus*, and *Candida kefyr* and the bacteria *Levilactobacillus brevis*, *Lacticaseibacillus paracasei*, *Lactobacillus helveticus*, *Lactobacillus kefiranofaciens*, *Lactiplantibacillus plantarum*, *Lentilactobacillus kefiri*, *L. lactis*, *S. thermophilus*, *Acetobacter lovaniensis*, *Acetobacter orientalis*, and *Leuconostoc mesenteroides* [55].

In addition, some LAB have been isolated from a pre-Hispanic beverage of Mayan origin from regions located in southwestern Mexico. Among them, we can mention pozol, a drink based on cocoa and nixtamalized corn. Strains of the bacterium *Weissella confusa* have been isolated from pozol, demonstrating that this drink represents an interesting source for the isolation of new potentially probiotic strains and their possible use in a functional food [56].

## 5. Microbial Biogenic Metabolites Derived from Fermentation

Among the major biogenic metabolites derived from fermentation produced by microorganisms are D-lactate, L-lactate, SCFAs, ammonia, amines, phenols, and indoles [57]. Additionally, a large number of fermentable carbohydrates that can reduce fermentation-derived metabolites within the gastric tract have been studied, demonstrating that functional food diets supplemented with probiotics can reduce the formation of ammonia, amines, phenolic, and indole compounds in different parts of the intestine, with more beneficial effects in the distal part of the colon [58].

The synergistic effects of fermentable carbohydrate, carbohydrate, and protein content and their relationship to intestinal health have been studied. This effect provides essential benefits to the growth performance of the individual due to the absorption of nutrients. We propose that foods containing fermentable carbohydrates, such as fruits like apples and pears, and vegetables, such as cabbage, broccoli, garlic, and beans, be considered main functional foods for their high content of macronutrients that function as symbiotics for the intestinal microbiota, including probiotic lactobacilli [59].

## 6. Mechanisms of Probiotic Functionality and Their Beneficial Effects

Several mechanisms of the functionality of probiotics have been studied, such as their support to the immune system. Once administered, the probiotic bacteria that enter the host orally will interact with the intestinal epithelial cells that line the microvilli of the intestine, associating with the immune cells associated with the lamina propria through Toll-like receptors, which induce the production of different cytokines and chemokines [60].

The production results in probiotic bacteria colonizing the gastrointestinal tract and selectively adhering to the cells, preventing the adhesion of pathogenic bacteria and the production of lactic acid, which lowers the pH of the microhabitat. These colonies have important advantages in the fight against many pathologies, leading to the proposal of a diet supplemented with probiotics mixing functional foods. Lactic acid bacteria are the diet’s main element, as in the cases of milk serums with added bacteria of the *Lacticaseibacillus casei* strain, yogurts produced from lactic cultures, the gels with *Bacillus coagulans* spores for renal patients, and the use of serums with different strains of *Lactococcus* as a dietary aid in the treatment of colon cancer [61].

### 6.1. Gastrointestinal Disorders

#### 6.1.1. Inflammatory Bowel Diseases (IBD)

One of the most common gastrointestinal diseases is ulcerative colitis (a type of IBD). This is an inflammatory disease that mainly affects the colon and rectum. Genetic factors, host immune system disorders, dysbiosis of the intestinal microbiota, and environmental factors contribute to the pathogenesis. Studies have shown that probiotics improve intestinal mucosal barrier function and immune system function and promote the secretion of anti-inflammatory factors, inhibiting the growth of harmful bacteria in the gut.

Fecal microbiota transplantation reduces intestinal permeability and, thus, disease severity by increasing the production of SCFAs, especially butyrate, which helps maintain the integrity of the epithelial barrier. The use of probiotic diets can also restore immune dysbiosis by inhibiting Th1 differentiation, T-cell activity, leukocyte adhesion, and inflammatory factor production. Probiotics and functional foods are increasingly used to treat colitis [62].

#### 6.1.2. Diarrhea

Probiotics are microorganisms that have been shown to be effective when added to a soft diet, such as gelatin or whey. They are often considered functional foods, as they contain strains of lactobacilli that confer a health benefit when consumed. Probiotics have been recommended during the symptomatology of diarrhea as they act as anti-pathogens, avoiding the prolonged use of antibiotics that irritate the intestinal mucosa [63].

#### 6.1.3. Food Allergy

Evidence is now emerging suggesting that the increase in food allergies is associated with functional and compositional changes in the gut microbiota. Microbiota-host interactions play a crucial role in the regulation of the immune system. The development of the gut microbiota and immune system in homeostasis occurs early in life. It is largely dependent on exposure to maternal microorganisms through natural vaginal delivery. In addition, the consumption of antibiotics during colostrum and breast milk intake by infants can alter intestinal homeostasis and significantly increase the risk of allergic diseases [64].

Functional foods supplemented with probiotics have been proposed, mainly natural tea-based beverages such as Baitang purple tea, containing anthocyanins that can serve as prebiotics [65]. Thus, changes in the quantity, diversity, or growth of microorganisms in the gastric tract affect oral tolerance through interactions of microbial molecules with pattern recognition receptors on immune cells and confer susceptibility to food-type allergies. In this context, products of the fermentation of insoluble fibers (e.g., SCFAs) by intestinal microorganisms have been shown to confer protective effects against food allergies. In recent years, probiotics have gained much attention as a preventive and therapeutic treatment for food allergies [66].

#### 6.1.4. *Helicobacter pylori* Infection

Infections of the gastrointestinal tract by bacteria are widespread. However, in some cases, there are complications in the treatment due to antibiotic resistance. This occurs in bacteria classified as multidrug-resistant; one of these is Helicobacter pylori, whose treatment is based on the application of third-generation antibiotics for the management of gastrointestinal disorders such as peptic ulcers and gastric cancer [67]. Due to the increased prevalence of *H. pylori* resistance to antibiotics, triple therapy with clarithromycin has ceased to work as a primary treatment, especially in some areas where local resistance to this antibiotic is higher than 20%. Alternative treatments for *H. pylori* eradication have been proposed. Some of them include novel or classical antibiotics in different combinations. Other therapies use probiotics such as lactobacilli and Bifidobacterium, which demonstrate their probiotic capacity as pathogen antagonists by releasing immunomodulators that stimulate the immune system. Also, a diet based on functional foods such as juices supplemented with probiotics and antibiotics to treat this infection [68].

#### 6.1.5. Lactose Intolerance

More than 60% of the human population has a reduced ability to digest lactose sugar due to low levels of lactase enzyme activity in their body. Probiotics are active bacteria or yeasts with constant metabolic activity that complement the gastrointestinal microbiota [69]. Studies have shown that probiotics exhibit several health-promoting properties, such as improving intestinal health, immune response, and lowering serum cholesterol. It has been shown that probiotic bacteria present in dairy products such as milk, yogurt, and fermented and non-fermented cheeses can serve to alleviate symptoms. Most LAB possess the enzyme lactase, which breaks down lactose, either contained in food or entering the host. Thus, the enzyme from probiotic bacteria helps to ameliorate the clinical pictures of lactose intolerance [70].

#### 6.1.6. Cancer

Cancer is a deadly neoplasm caused by the proliferation of abnormal cells that continuously replicate uncontrollably and remains one of the leading causes of disease and death. There is currently no definitive or accurate treatment. The safety and stability of standard chemotherapeutic drugs and synthetic agents used to treat cancer are very aggressive to the human body. They cause sequelae that systemically damage normal organs and cells in the human body [71]. These drug treatments affect the quality of life or contribute to the development of drug resistance. Metabolically active or inactive probiotics and their metabolites (SCFAs, protein inhibitory compounds, polysaccharides, nucleic acid, and ferrichrome) are a natural and effective treatment. This treatment has been proposed as an alternative cancer prevention and treatment due to the content of alternative biomolecules and biotherapeutics against cancer since they release molecules with antioxidant capacity that inhibit the proliferation of cancer cells [72].

## 7. Discussion and Conclusions

Current advances in the gut microbiota field bring new frontiers of functional foods, nutraceuticals, and probiotics research to the future. Indeed, in recent years, interest in this area of study has continued to increase, leading to some important advances discussed in this review. The new technologies, mechanisms, and applications that are currently being explored offer the possibility of changing the scientific knowledge, as well as the dietary and health benefits of these interventions. However, despite this progress, many questions remain to be answered, especially regarding the immune response generated in the host by probiotics. As we have reviewed, probiotics have a wide application in the medicinal area, in the veterinary field, in the industrial food area, and in the technology sector to produce nutraceuticals and drugs; however, it is still necessary to study various models of isolation of probiotic bacteria, as well as different isolation sources.

In the development of probiotic foods intended for human consumption, different strains of LAB (such as *Lactococcus*, lactobacilli, and *Streptococcus*), as well as some strains of *Bifidobacterium* and yeasts, have been traditionally used in the manufacture of fermented dairy products and have GRAS (Generally Recognized As Safe) status. However, although the probiotic market is dominated by fermented dairy products, several screening studies have now been conducted on different non-dairy matrices (such as Korean, Indian, African, or Mexican traditional fermented foods) to select new probiotic strains for the food industry. Such studies have highlighted the probiotic potential of novel non-human, non-dairy LAB strains and opened new applications beyond dairy products. For example, LAB isolated from sourdough or traditional beverages may be a good source of potentially beneficial and useful LAB.

In conclusion, as we have explored, probiotic microorganisms can be isolated from many sources, mainly from food, which leads to propose their future use in the application of functional foods to improve our quality of life.

## Data Availability

Not applicable.
